# Return to work after a workplace-oriented intervention for patients on sick-leave for burnout - a prospective controlled study

**DOI:** 10.1186/1471-2458-10-301

**Published:** 2010-06-02

**Authors:** Björn Karlson, Peter Jönsson, Birgitta Pålsson, Gunnel Åbjörnsson, Birgitta Malmberg, Britt Larsson, Kai Österberg

**Affiliations:** 1 Division of Occupational and Environmental Medicine, Department of Laboratory Medicine, Lund University, Lund, Sweden; 2 Division of Rehabilitation Medicine, Department of Clinical and Experimental Medicine, Faculty of Health Sciences, Linköping University, Linköping, Sweden

## Abstract

**Background:**

In the present study the effect of a workplace-oriented intervention for persons on long-term sick leave for clinical burnout, aimed at facilitating return to work (RTW) by job-person match through patient-supervisor communication, was evaluated. We hypothesised that the intervention group would show a more successful RTW than a control group.

**Methods:**

In a prospective controlled study, subjects were identified by the regional social insurance office 2-6 months after the first day on sick leave. The intervention group (n = 74) was compared to a control group who had declined participation, being matched by length of sick leave (n = 74). The RTW was followed up, using sick-listing register data, until 1.5 years after the time of intervention.

**Results:**

There was a linear increase of RTW in the intervention group during the 1.5-year follow-up period, and 89% of subjects had returned to work to some extent at the end of the follow-up period. The increase in RTW in the control group came to a halt after six months, and only 73% had returned to work to some extent at the end of the 1.5-year follow-up.

**Conclusions:**

We conclude that the present study demonstrated an improvement of long-term RTW after a workplace-oriented intervention for patients on long-term sick leave due to burnout.

**Trial registration:**

Current Controlled Trials NCT01039168.

## Background

Work stress has been proposed as a major cause of health problems and sick leave in the European Union countries [1]. Since the late 1990 s, long-term sick leaves have increased rapidly in Sweden, particularly due to mental illnesses, and constitute for the period 2003-2008 around 35% of all long-term sick leaves for white-collar employees in Sweden [2]. These sick leaves often seem to be related to long-term work stress and exhaustion, similar to the core dimension in various definitions of burnout.

There is a lack of established and evaluated programs for treating these patients in primary and occupational health care. This situation may lead to prolonged sick leave or even loss of employment and disability pension.

Only a few controlled studies evaluating treatment programs for persons on sick leave due to work stress-related mental illness have been published [3,4]. As a rule, the interventions studied were oriented towards the individual and were of the cognitive-behavioural therapy (CBT) type, aiming to improve stress management. Typically, the effects on sick leave and return to work (RTW), as well as on symptoms, were marginal [3,5]. To our knowledge, only one study has involved workplace interventions, showing a clearly favourable effect on RTW when added to a brief CBT intervention [6].

Thus, to facilitate RTW, workplace-oriented interventions seem promising for several reasons. Work-related burnout has been suggested to be a consequence of a long-term mismatch between the person's abilities or expectations and the job's characteristics [7]. This mismatch may remain when the person returns to work, perpetuating the stressor. Even though interventions directed towards the individual may improve the ability to cope with stress or to solve problems, they may not be sufficient to facilitate RTW. Appropriate changes in the work environment or the individual's work situation, providing an improved job-person match, may be required to shorten the duration of the sick leave [8]. One specific contributing factor to long sick leaves may be insufficient contact between the patient and the supervisor [9]. For the patient, lack of contact may reinforce a sick role, by distancing him or her from concrete planning of how to return to work. For the employer, a sick leave of unknown duration requires adaptation, possibly leading to a decreased incentive to prepare for the patient's RTW. An additional benefit of organisational intervention is the potential of primary prevention for other employees at risk.

The present study specifically aimed to evaluate the effect on return to work of a workplace intervention with patients being treated for burnout. The intervention was intended to reduce job-person mismatch through patient-supervisor communication. The hypothesis was that the intervention group would show a more favourable outcome than a control group with respect to RTW.

## Methods

### Participants with burnout

Participants were recruited in co-operation with regional social insurance offices (RSIOs) in the two southern counties of Sweden. Recruitment started in November 2003, and the last participant was invited in October 2006. The basic criteria for inclusion were employment, sick-listing at least half-time for 2-6 months from a previously healthy state, and having an International Classification of Diseases (ICD-10) diagnosis within the F43 category (reaction to severe stress, and adjustment disorders, except post-traumatic stress disorder (F43.1)), due to predominantly work-related stressors, apart from severe conflicts or bullying. Social insurance officers identified all new cases from a register of ongoing sick-listings and checked the criteria of relationship between sick-listing and work stress, using information from the person's file. Those with a sick-leave related to private life, were excluded. However, to maximize the potential participation rate, persons with an uncertain relation between work stress and sick leave were included. A list of possible participants was sent to the research group, who informed the identified persons by letter about the project, followed up with a phone call to invite them to participate.

After acceptance, an initial screening for inclusion criteria was carried out by way of a questionnaire about the work situation (QPSNordic) [10], and an interview about the process leading to sick leave, with particular focus on the work situation and the relationship between work stress and sick leave. If no obvious obstacles to inclusion were found, patients who gave informed consent underwent a one-day close examination at the Occupational and Environmental Medicine Clinic, Lund University Hospital, Lund, Sweden. This comprised assessment by a senior physician, a psychologist, and a social worker. The examination included a medical workup, including lab tests, a structured interview screening for psychiatric illness, the Primary Care Evaluation (Prime-MD) [11], and an assessment of whether the patient met the criteria for "exhaustion disorder" suggested by the Swedish National Board of Health and Welfare [12], which require identifiable stressors during the past six months, accompanied by a number of symptoms. Moreover, we conducted an in-depth interview of the course of events leading up to exhaustion and recorded the patient's expectations of changes necessary to facilitate RTW. The patient also responded to a second set of questionnaires, reflecting mental distress, depression, and burnout (Table 1). His or her sick-listing doctor was informed about their participation in the project, but was otherwise not involved in the project.

**Table 1:** Baseline characteristics of the intervention group (n = 74)

Characteristic	*n *(%)	mean (SD)
Diagnoses		
Exhaustion disorder only^a^	28 (38)	..
Exhaustion disorder + depression or anxiety^b^	36 (49)	..
Exhaustion disorder + somatic disease	2 (3)	..
No exhaustion disorder	8 (11)	..

Treatment		
Medication		..
Antidepressants	19 (26)	..
Anxiolytics or sleeping pills	9(12)	..
Medication for somatic disorders^c^	21 (28)	..
No medication	35 (47)	..
Psychotherapy	41 (56)	..
Physiotherapy	8 (11)	
No treatment at all	21 (28)	..

MBI-GS	..	
Exhaustion score		4.6 (1.2)
Cynicism score		2.7 (1.4)
Professional efficacy score		4.4 (1.1)

SCL-90 subscales		
Anxiety	..	1.7 (0.8)
Somatisation	..	1.3 (0.8)
Depression	..	2.0 (0.9)

BDI classification	..	19.3 (9.1)
Severe depression	10 (14)	..
Moderate depression	36 (49)	..
Slight depression	16 (22)	..
Minimal depression	12 (16)	..

^a^According to criteria for "exhaustion disorder" (F43.8A) suggested by the Swedish National Board of Health and Welfare[12]^b^Psychiatric diagnoses according to interview with Prime-MD = Primary Care Evaluation of Mental Disorders[11]^c^Mostly antihypertensive, antihyperlipidemic, and antidiabetic drugs, beta blockers, vitamin or mineral supplements, or weight control drugs.

MBI-GS = Maslach Burnout Inventory--General Survey[13]. SCL-90 = Symptom Checklist 90[16] BDI = Beck Depression Inventory (BDI)[17]

**Figure 1:**
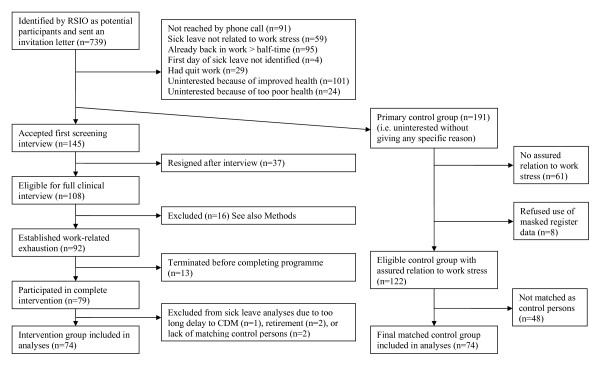
**Flow diagram showing the recruitment of intervention and control groups**.

Of a total of 739 people who were sent the information letter, 108 who agreed to participate were considered eligible for full clinical examination (Figure 1). Of these, 16 were excluded because their symptoms had other possible causes, such as other disease, strain stemming from private life, or relapse of mental illness without clear relationship to workload. After excluding participants who did not complete the intervention, 74 persons (59 women) with a mean age of 46.6 years (SD = 9.1) were finally included in the analyses. In all, 89% met the criteria for exhaustion disorder [12]. Forty-six percent received a diagnosis of depression and/or anxiety in Prime-MD. Exhaustion was verified with Maslach Burnout Inventory--General Survey (MBI-GS) [13], showing a mean exhaustion score of 4.6 (SD 1.2), compared with mean scores in the 1.2-1.9 range found in Scandinavian populations [14,15]. Self-ratings of mental distress, Symptom Checklist-90 (SCL-90) [16], and Beck Depression Inventory (BDI) [17] also showed elevated levels (Table 1). The group represented a wide range of occupations in blue- and white-collar jobs, in private as well as public sector, and had varying levels of education (51% had studied at university, 36% at upper secondary school, and 12% had 9-year compulsory schooling).

### Control group

The original intent was to randomise participation among the persons listed by the RSIOs by inviting every second person to participate in the intervention and using the remainder as controls. However, to get enough participants, we had to invite all persons on the lists. This necessitated using as our primary control group the 191 persons who had simply been uninterested in participating and had not given any specific reason. In contrast to the intervention group, as controls were selected only those persons for which the relation between work stress and sick leave had already been confirmed based on the RSIO data (n = 130). They were sent an information letter stating that their sick-listing data registered at the RSIO would be used in the research project with personal identities masked to the research group. Eight persons denied the use of their data, resulting in 122 controls, of whom 74 (56 women) were matched as final controls based on sick-leave data (see method below). Their mean age was 46.1 years (SD = 11.1).

### Intervention procedure

Based on questionnaire replies and interview data, the course of events leading up to exhaustion, and the patient's views of the changes needed to facilitate RTW, an outline of the patient's perspective was compiled. Next, with permission of the patient, his or her nearest supervisor was interviewed at the workplace, responding to the same questions on perceived main causes of the employee's sick leave and changes necessary to facilitate RTW.

Then followed the core intervention--a convergence dialogue meeting (CDM). The purpose of the CDM was to initiate a dialogue between the patient and the supervisor to find solutions to facilitate RTW. The CDM was carried out at the workplace, with two team members who had examined the patient. The CDM started with the team members' summary of the perspectives of the patient and the supervisor, highlighting their agreements and disagreements on the causes for the sick-leave and on necessary changes for facilitating RTW. The main focus was on solutions and suggested changes, that is, striving for converging perspectives and goals between supervisor and patient. The CDM typically lasted for about 1.5 hours, and usually resulted in agreements about short- and long-term solutions. A concluding summary of the results of the clinical examination and the CDM was sent to the social insurance office, the patient, and his or her sick-listing doctor.

Some weeks later, patients were invited to a half-day group seminar, together with 4-6 other patients who had undergone the same programme. The seminar consisted of lectures followed by discussions on the topic work-related stress. Similar seminars were arranged for involved supervisors. The seminars had preventive aims--for the patients to reflect on how to prevent a new, similar occurrence of sick leave, and for the supervisors on how to prevent work stress-related sick leaves among their employees.

### Methodological considerations

#### Theoretical base

A theoretical base for the intervention was to apply the mismatch perspective, originally used as a causal model for the development of burnout [7], to an intervention perspective. This was done by identifying ways to achieve an improved match between the patient's abilities, expectations, and needs and the characteristics of the job. This perspective was a strategic choice, focusing on the fit between the job and the person, which was assumed to promote a constructive patient-supervisor communication, compared to a focus on causes for sick-leave. The six dimensions of the model suggested having a causal role in the development of burnout--problems in the areas of demand, control, reward, community, fairness, and values--were used to conceptualise and assess the job-person (mis)match.

#### Timing of the intervention

It could, on the one hand, be argued that a contact with the workplace to initiate an occupational rehabilitation planning process would be meaningless, and possibly harmful, at too early a stage of the sick leave, since the patient might not be well enough to make plans for the future. On the other hand, it could be argued that a workplace contact early on in the sick leave phase would sustain the contact. As the risk of developing very long sick leaves often increases after about two months of sick leave, we chose to start the intervention at about that time, considering that to be most cost effective.

#### Recruitment and selection of participants

To recruit participants among consecutive patients at health care units might introduce uncontrolled selection bias due to, for example, the patients' trajectory through the health care system. Such selection bias was avoided by co-operating with RSIOs, giving us access to all consecutively sick-listed persons within a defined geographical region who fulfilled the basic inclusion criteria. This method, by not restricting the selection base to, for instance, a specific company, health care unit, or socio-economic group, also offered results that could be generalised to a wider population.

The selection of a control group and control conditions is a delicate matter in prospective intervention studies. Offering a control condition that is perceived as less attractive than the intervention may lead to dropout, with risk for differential attrition. Similarly, the control group may acquire additional desired treatment in some other way, with the consequence of diminishing group contrast. We chose not to offer any other type of control intervention, by letting both groups, besides our intervention, get "care as usual" (CAU), which could vary depending on local opportunities at their workplace and place of residence. Moreover, the control group was passively followed through sick leave register data only, in order to avoid any unintended "light" intervention that might have been brought about through examinations and interviews.

### Sick leave data

From the first day of the particular sick leave episode that gave rise to project participation, the RSIO listed all following episodes until August 2009. The degree of sick leave (0, 25, 50, 75, or 100% of ordinary working time) each week was entered as variables (i.e. week x: 50%; week y: 75%, etc.). A week was defined as four days or more. The aim was to monitor each person's sick leave during at least one year following the CDM.

### Data management and matching of control group

The week of the CDM was defined as week zero (W0). Sick leave data for 80 weeks after W0 was entered into the database.

For each participating patient we calculated the number of weeks that elapsed before the CDM, counted from the first day on sick leave and the mean sick leave percent. This constituted the base for the matching of the control group. For example, a patient who had waited 35 weeks to attend the CDM, during which she or he had a mean of 75% sick leave, was matched with a control person with corresponding data.

The control participants were randomly assigned a number and then sorted in ascending order. The first control person who fulfilled the criteria of the first patient was chosen, that is, 35 weeks' initial sick leave of 75%, in the example above. This procedure was continued until every patient had been matched to a control participant.

### Statistics

The analyses were based on dichotomised data, that is, RTW 25% or more (YES) vs. not back to work (NO), using the generalised estimating equation (IBM SPSS Statistics 18.0) to examine omnibus effects with GROUP (intervention group and controls) as between subject factor and WEEKS (every 10th week from W0 to W80, i.e. 9 levels) as repeated factor, with RTW (YES, NO) as dependent variable. Logit link function and auto regressive (AR(1)) correlation matrix were used. For the repeated factor, polynomial contrasts were analysed. Significance test were performed with Wald χ^2 ^(α = .05).

For descriptive purposes, raw data comprising 0, 25, 50, 75, and 100% sick leave (SICK LEAVE) were analysed with Pearson χ^2 ^exact tests with SICK LEAVE and GROUP as factors, separately for each week.

### Ethics

All participants gave written informed consent to participate in the study, and the study protocol was approved by the Ethics Committee of Lund University (LU 784-03).

## Results

### Total group

#### Return to work

**Figure 2:**
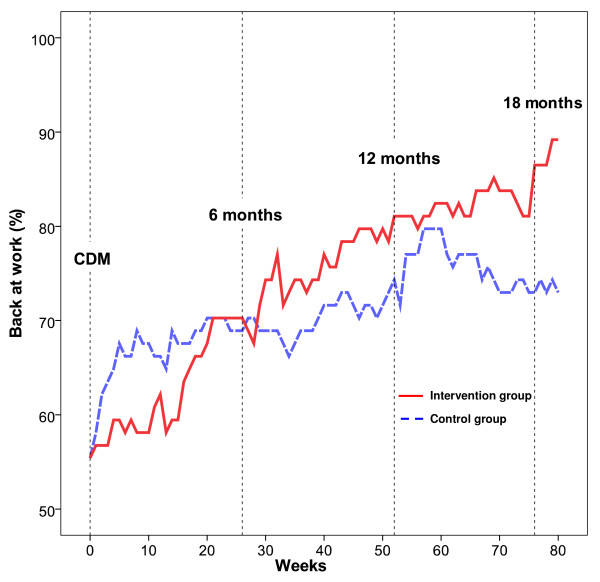
**Proportions having returned to work on at least 25% during the study period**. Graphs show the development of return to work, as proportions (percentages) of the intervention and control groups, from the time of the convergence dialogue meeting (CDM) until week 80.

The omnibus test showed a significant main effect of WEEKS [χ^2 ^(8) = 34.57, p < .0001], with a linear contrast [χ^2 ^(1) = 31.36, p < .0001] and a quadratic contrast [χ^2 ^(1) = 6.95, p = .008]. A GROUP × WEEKS interaction effect [χ^2 ^(8) = 21.52, p = .006] indicated that the development of sick leave over time differed between groups. Separate analyses for GROUP showed a linear contrast for the intervention group [χ^2 ^(1) = 26.07, p < .0001], indicating a rather stable RTW across weeks. The control group showed a more complex pattern of RTW: A linear contrast [χ^2 ^(1) = 6.87, p = .009] corresponded to an increasing trend of RTW, but a quadratic contrast [χ^2 ^(1) = 7.48, p = .006] showed that RTW was not that stable, that is, first an increase and then a decrease of RTW. Finally, a contrast of the fourth degree [χ^2 ^(1) = 4.42, p = .035] matched the pattern in the control group as depicted in Figure 2; a quick initial increase from week 0 to week 10, that declined until week 30 when a second increase occurred that reached its peak at week 60, after which RTW decreased until week 80.

Exploring AGE as a covariate did not reveal any significant interaction. Including GENDER as a factor in the model did not result in a significant interaction with GROUP and WEEKS. However, there was a significant GENDER*WEEKS interaction [χ^2 ^(8) = 15.87, p = .045]. Separate analyzes indicated that women showed a linear increase in RTW from 53% at week 0 to 81% at week 80 [linear contrast: χ^2 ^(1) = 29.52, p < .0001]. Men initially returned to work more rapidly; from 64% at week 0 to 85% at week 30, a result that was constant until week 60 when a slight decrease occurred that ended with 82% RTW at week 80 [quadratic contrast: χ^2 ^(1) = 10.17, p = .001].

#### Raw data

At the time of the CDM, the two groups' sick leaves were similarly distributed. From ten weeks and onwards the control group tended to be dichotomised, that is, most of the group were either back to work full time or on sick leave full time, while the intervention group showed more evenly distributed sick leave data across the steps of 0, 25, 50, 75, 100% (Table 2). Basically, the control group maintained this dichotomised pattern to the end of the study period (week 80), at which time 64% had fully returned to work and 27% were on 100% sick leave. The number of participants in the intervention group on 100% sick leave decreased to about 11% at week 80, while 64% were fully back in work, and the rest worked 25-75%.

**Table 2:** Sick leave in the intervention and control groups as a function of weeks.

	Sick leave (%)					χ^2^	*p*
			
	0	25	50	75	100		
	Week 0						
Control	20.3 (15)	10.8 (8)	21.6 (16)	2.7 (2)	44.6 (33)	5.83	.22
IG	9.5 (7)	12.2 (9)	24.3 (18)	9.5 (7)	44.6 (33)		

	Week 10						
Control	41.9 (31)	13.5 (10)	10.8 (8)	1.4 (1)	32.4 (24)	16.62	.001
IG	14.9 (11)	12.2 (9)	28.4 (21)	2.7 (2)	41.9 (31)		

	Week 20						
Control	55.4 (41)	6.8 (5)	8.1 (6)	0 (0)	29.7 (22)	22.45	<.001
IG	23.0 (17)	13.5 (10)	25.7 (19)	5.4 (4)	32.4 (24)		

	Week 30						
Control	51.4 (38)	6.8 (5)	10.8 (8)	0 (0)	31.1 (23)	16.38	.002
IG	28.4 (21)	14.9 (11)	24.3 (18)	6.8 (5)	25.7 (19)		

	Week 40						
Control	56.8 (42)	6.8 (5)	6.8 (5)	1.4 (1)	28.4 (21)	8.77	.061
IG	41.9 (31)	10.8 (8)	20.3 (15)	4.1 (3)	23.0 (17)		

	Week 50						
Control	56.8 (42)	5.4 (4)	8.1 (6)	1.4 (1)	28.4 (21)	8.45	.074
IG	44.6 (33)	13.5 (10)	16.2 (12)	5.4 (4)	20.3 (15)		

	Week 60						
Control	64.9 (48)	8.1 (6)	5.4 (4)	1.4 (1)	20.3 (15)	5.16	.275
IG	54.1 (40)	10.8 (8)	10.8 (8)	6.8 (5)	17.6 (13)		

	Week 70						
Control	62.2 (46)	4.1 (3)	6.8 (5)	0 (0)	27.0 (20)	8.06	.078
IG	56.8 (42)	8.1 (6)	16.2 (12)	2.7 (2)	16.2 (12)		

	Week 80						
Control	63.5 (47)	4.1 (3)	5.4 (4)	0 (0)	27.0 (20)	11.64	.013
IG	63.5 (47)	6.8 (5)	16.2 (12)	2.7 (2)	10.8 (8)		

Distributions of sick leave (percentages and number of persons) at week 80 in the intervention group (IG) and the control group (Control) throughout the study period.

### Participants with at least 75% sick leave at week 0

#### Return to work

This analysis too, showed a significant main effect of WEEKS [χ^2 ^(8) = 68.754, p < .0001], together with a linear contrast [χ^2 ^(1) = 69.41, p < .0001], and a quadratic contrast [χ^2 ^(1) = 18.00, p = .0001]. The WEEKS × GROUP interaction was also significant [χ^2 ^(8) = 20.28, p = .009]. Separate analyses for GROUP revealed for both groups a linear contrast [intervention group: χ^2 ^(1) = 44.95, p < .0001; control group: χ^2 ^(1) = 27.03, p = .0001] and a quadratic contrast [intervention group: χ^2 ^(1) = 9.59, p = .002; control group: χ^2 ^(1) = 10.47, p = .001]. In the control group the 4^th ^degree contrast was also significant [χ^2 ^(1) = 5.41, p = .015]. Thus, the patterns of sick leave over the weeks were similar to the results found in the total group.

#### Raw data

After 80 weeks, 83% of the intervention group were back to work to some extent, compared to 57% of the control group (Table 3). Hence, even among subjects who may be considered to have been suffering from more severe burnout, the intervention group had a proportional advantage in RTW compared to the control group, similar to the finding in the total group.

**Table 3:** Sick leave at week 80 for the subgroups having ≥75% sick leave at week 0.

	Sick leave (%)					χ^2^	*p*
			
	0	25	50	75	100		
	Week 80						
Control	42.9 (15)	5.7 (2)	8.6 (3)	0 (0)	42.9 (15)	8.02	.079
IG	47.5 (19)	10.0 (4)	20.0 (8)	5.0 (2)	17.5 (7)		

Distributions of sick leave (percentages and number of persons) at week 80 in the subsamples of the intervention group (IG) and the control group (Control) that were on at least 75% sick leave at the time of the convergence dialogue meeting.

## Discussion

The aim of this study was to evaluate the effect of a workplace intervention on the sustainable return to work of persons on long-term sick leave for clinical burnout. The hypothesis of a more favourable RTW in the intervention group, compared to a matched control group receiving only "care as usual", was confirmed. This is in line with the results of the only previous study that, to our knowledge, has evaluated workplace interventions in a similar group [6].

As shown in Figure 2 the intervention group showed a rather stable and linear return to work, from about 55% at the time of the convergence dialog meeting to 89% at week 80 after the meeting. The control group, also starting with a 55% RTW, ended up with a 73% RTW. Interestingly, the pattern of RTW across weeks was somewhat unstable in latter group, showing a steep increase of RTW during the first 10 weeks, and a sudden decrease of RTW over the last 20 weeks. The two groups showed different distribution patterns of RTW across weeks. The control group showed an increasing tendency to form a bimodal distribution of either being on full sick leave or having fully returned to work. In the intervention group, RTW was more common with gradual work resumption, a strategy commonly agreed upon in the convergence dialogue meeting. However, the number of persons having fully returned to work was equal in the two groups at the end of the follow-up. The RTW in the groups seemed to converge after six months and after about a year, after which they separated again in favour of the intervention group. The reason for this temporarily converging pattern can only be speculated about. A longer follow-up is planned and may shed light on the relevance of such fluctuations in RTW patterns.

The reason for the tendency towards a bimodal distribution of RTW in the control group is not clear. For the control group, we only had register data on sick leave, so we do not know what actions, if any, were taken to instigate RTW in this group. Speculatively, the bimodal distribution might have to do with a greater heterogeneity in basic group characteristics, such as clinical status or work ability. However, in the selection of eligible control persons, those declining to participate due to either having recovered or feeling too ill, were not included. Thus, there is no reason to believe that the control group was extreme in either direction. The question may also be raised whether the poorer RTW in the control group may have been due to having fewer rehabilitation resources available in their CAU, though it seems more probable that lacking availability of such resources would have increased the likelihood of accepting the intervention offered, rather than rejecting it.

The higher proportion of *full-time *RTW in the control group during the first year might, on the one hand, be due to different handling of their sick-listing process by the social insurance offices, such that they felt forced to return to work prematurely. If so, an increased risk for later relapses into new sick leaves would have been expected in this group, which in fact did not occur. On the other hand, in the intervention group it was not uncommon to have an unintended delay in the work resumption process, because of problems contacting or making appointments with the patient's supervisor, and thus concluding the CDM. Also, the patients' doctors sometimes waited for the intervention to be completed before suggesting resumption of work.

Blonk et al. [6] discussed the possibility that a partial RTW may promote a later full RTW. Since gradual RTW often formed an intrinsic part of the total strategy for RTW that developed during the CDM, it was not possible to evaluate the separate effect of gradual RTW *per se*. Possibly, gradual work resumption may be one of the more important components negotiated during the CDM. One of the major aspects of gradual RTW might be to prevent exclusion from the labour market, as illustrated by the substantially lower proportion of persons in full-time sick leave in the intervention group at the end of the study period.

### Relation to previous studies

When comparing the results with other similar studies, cross-national comparisons must be made with caution due to differences in social security systems and cultures. In all three previous Swedish studies the participants had been on sick leave for a longer time prior to inclusion in the studies (mean range 9-24 months), compared to our group. Moreover, only individual treatment programs were evaluated. After group cognitive-behavioural therapy combined with Qigong, compared to Qigong only, more than half the burnout patients on long-term sick leave in both programmes showed at least a partial RTW after one year, while 40% were still on full-time sick leave [4]. A stress management intervention compared to CAU, in women with work stress-related sick leave in a company health care unit, resulted in only 40% RTW for both groups, as long as 5 years after the intervention [18]. When comparing a CBT training program, a physical activity program, and CAU for patients on 1-24 months' sick leave for stress-related diagnoses, a 41% at least part-time RTW was found one year after the intervention, with only minor group differences [19].

Three studies are from the Netherlands, one showing a quicker RTW for an intervention group receiving a CBT intervention compared to CAU, with all participants of both groups back at work after 1 year. In this study, carried out in one company, participants were enrolled after only two weeks of sick leave [20]. In a self-employed, predominantly male group, CBT was compared with a combined individual and workplace intervention and CAU as control condition. The combined intervention, starting a few weeks to a few months after the first sick day, gave a clearly superior RTW compared to the CBT and control conditions, although the actual RTW proportions were not reported [6]. Recently, the three conditions of a CBT-based stress management training, either in group or individually, vs. CAU, were compared in clinical samples of patients with work-related stress who had been on sick-leave for between 2 weeks and 6 months, showing no difference in symptom reduction or sick leave between the conditions during a 10-month follow-up [3]. The authors concluded that CBT interventions are not effective in treating patients with work-related stress. In a Danish study [21] using a similar methodology as van der Klink [20] for persons on sick leave for mental distress, about 80% of the intervention group as well as of a CAU control group returned to work within one year.

Thus, our study gave results clearly superior in terms of RTW, compared to all previous Swedish studies. The main differences are that we intervened in an earlier phase of the sick leave history, with an intervention directed towards the workplace that had the goal of facilitating RTW. These aspects of timing and type of intervention are factors that have been suggested in several studies for future improvement of results [3,4,6,19]. A longer duration of sick leave prior to being included in the studies was in one study shown to be related to a higher future sick leave [4]. All Dutch studies involved participants sooner after the initiation of sick leave than the Swedish studies, although the proportion of RTW was not explicitly reported in most Dutch studies, making results hard to compare. Only van der Klink [20] clearly reported the proportion of RTW, which was even more favourable than in our study. Carried out within one company, with enrolment in the study only two weeks after the first sick day, the study included persons who probably spontaneously would have returned to work.

However, because our control group also had a better proportion of RTW than in the previous Swedish studies, short time to intervention may not be the only explanation. One possible contributing factor to the divergent findings may be that the selection procedures differed across studies and were confounded by length of sick leave. Recruitment from health care units may have implied varying durations of sick leave prior to intervention, and possibly differing clinical severity. Our selection method, using RSIO registry data, gave a fairly homogeneous sick-listing time prior to enrolment in the study, good control over the participation rate, and some knowledge of non-participation reasons. Because the participation rate was low also with our recruitment procedure, some self-selection bias may still have occurred. Nonetheless, a review of the RTW proportion among those declining participation due to having recovered or being back at work showed nearly 80% on the job already at week 0, with only a marginal increase of RTW at week 80, indicating that our participants were not disproportionately healthy.

### Clinical severity and RTW

A probable variation of clinical severity across previous studies is hard to evaluate, since the clinical state was measured and described in different ways. Most studies found no association between improvement of complaints and RTW, which indicates that an increased well-being in itself is not always sufficient for RTW to occur. Yet, some degree of improved well-being is a likely prerequisite for a successful initiation of sustainable work resumption. In one study, a high burnout score was negatively related to RTW at follow-up [4], and in another study, the patients' expectations of their health and perceived cognitive resources was associated with a successful RTW [19]. Despite these findings, the sub-group who were on sick leave 75-100% at week 0, presumably representing more severe cases with higher risk of long-term sick leave, showed the same proportional advantage in RTW by having received the workplace intervention compared to the control group (83% and 57%, respectively). This finding suggests that the effectiveness of the workplace intervention is not restricted to milder cases of exhaustion.

### Limitations

There are some limitations to our study. The selection method gave a poor knowledge of the basic characteristics of our control group, limiting the possibility to analyse possible self-selection bias. However, as previously discussed, the analyses actually carried out did not give reason to assume that the participants were either obviously healthier or in a worse condition than the control group. Still, other self-selection effects cannot be ruled out, for example, selection of those having a more positive work situation from the start, or a better contact with their supervisors, but selections working in the opposite direction might also have occurred, for example, those in urgent need of help because of a severe work situation. Similarly, there may have been a number of reasons for the lack of motivation to participate among those used as control persons. Some reasons may be related to a reduced probability to return to work, and some may be related to expectations to be able to return to work without the offered intervention. The selection bias that has not been possible to fully control in the study calls for some caution about the conclusions drawn.

### Effective elements of the intervention

Although it is beyond the scope of this paper to analyse predictors for successful RTW within the intervention group, some speculations can be made about which were the effective elements of the intervention. In addition to actual changes in the work situation, the intervention implied feedback and clarification of how the work situation had been, which could make it more comprehensible. The dialogue with the supervisor may have helped to diminish negative feelings and cognitions associated with the workplace and the events preceding the sick leave, thereby, creating a readiness for RTW. Often the CDM also resulted in agreements for improved and regular communication between the supervisor and the patient, which has previously been shown to be effective [9]. It is also possible that the focus put on the patient may have highlighted the patient's situation for the surrounding actors (i.e. the supervisor, the doctor, and the RSIO through our summary) and yielded beneficial handling of his or her work rehabilitation.

### Applicability

Because the subjects span a large geographical area and include urban and rural settings, public and private employees, small to large companies or employers, and a variety of socio-economic groups and occupations, the results can be broadly generalised beyond the study population. The ability to generalise the findings is also high with respect to age, as a supplementary analysis did not show any differential RTW effects in relation to age. The applicability with respect to gender is slightly more uncertain, as men seemed to return to work more rapidly than women in both groups. Although this may really be the case, the effect size may be over-estimated due to the small male sample size.

## Conclusions

We conclude that the present study demonstrated that RTW was improved after a workplace-directed intervention, for patients on long-term sick leave for burnout.

## Competing interests

The authors declare that they have no competing interests.

## Authors' contributions

BK drafted the manuscript. All authors but PJ participated in the design of the study and acquisition of data. KÖ in addition helped to draft the manuscript. PJ performed the statistical analyses and helped to draft the manuscript. All other authors contributed with minor comments to the manuscript, and all authors read and approved the final manuscript.

## Pre-publication history

The pre-publication history for this paper can be accessed here:

http://www.biomedcentral.com/1471-2458/10/301/prepub

## References

[B1] Parent-Thirion A Fernández Macias E Hurley J Vermeylen G Fourth European Survey on Working Conditions 2005 2007 Luxembourg: Office for Official Publications of the European Communities

[B2] Hälsobarometern 2008.06-2009.05 http://www.alecta.se/upload/Hälsobarometern/Filer/2009/HB_2009.pdf

[B3] de Vente W Kamphuis JH Emmelkamp PM Blonk RW Individual and group cognitive-behavioral treatment for work-related stress complaints and sickness absence: a randomized controlled trial J Occup Health Psychol 2008 13 3 214 231 10.1037/1076-8998.13.3.214 18572993

[B4] Stenlund T Ahlgren C Lindahl B Burell G Steinholtz K Edlund C Nilsson L Knutsson A Slunga Birgander L Cognitively Oriented Behavioral Rehabilitation in Combination with Qigong for Patients on Long-Term Sick Leave Because of Burnout: REST-A Randomized Clinical Trial Int J Behav Med 2009 19148765 10.1007/s12529-008-9011-7

[B5] Nieuwenhuijsen K Verbeek JH de Boer AG Blonk RW van Dijk FJ Predicting the duration of sickness absence for patients with common mental disorders in occupational health care Scand J Work Environ Health 2006 32 1 67 74 16539174 10.5271/sjweh.978

[B6] Blonk RWB Brenninkmeijer V Lagerveld SE Houtman ILD Return to work: A comparison of two cognitive behavioural interventions in cases of work-related psychological complaints among the self-employed Work & Stress 2006 20 2 129 144

[B7] Maslach C Leiter MP The truth about burnout 1997 San Francisco: Jossey Bass

[B8] Nieuwenhuijsen K Verbeek JH Siemerink JC Tummers-Nijsen D Quality of rehabilitation among workers with adjustment disorders according to practice guidelines; a retrospective cohort study Occup Environ Med 2003 60 Suppl 1 i21 25 10.1136/oem.60.suppl_1.i21 12782743 PMC1765726

[B9] Nieuwenhuijsen K Verbeek JH de Boer AG Blonk RW van Dijk FJ Supervisory behaviour as a predictor of return to work in employees absent from work due to mental health problems Occup Environ Med 2004 61 10 817 823 10.1136/oem.2003.009688 15377767 PMC1740675

[B10] Lindström K Elo A-L Skogstad A Dallner M Gamberale F Hottinen V Knardahl S Ørhede E User's Guide for the QPSNordic. General Nordic Questionnaire for Psychological and Social Factors at Work TemaNord 2000 Copenhagen: Nordic Council of Ministers

[B11] Spitzer RL Williams JB Kroenke K Linzer M DeGruy FV Hahn SR Brody D Johnson JG Utility of a new procedure for diagnosing mental disorderin primary care. The PRIME-MD 1000 study J Am Med Assoc 1994 272 22 1749 1756 10.1001/jama.272.22.1749 7966923

[B12] Socialstyrelsen Utmattningssyndrom (Exhaustion disorder) 2003 Stockholm: Bjurner & Bruno

[B13] Maslach C Jackson SE Leiter MP MBI: The Maslach Burnout Inventory: Manual 1996 Press CP. Palo Alto

[B14] Ahola K Honkonen T Isometsä E Kalimo R Nykyri E Koskinen S Aromaa A Lönnqvist J Burnout in the general population. Results from the Finnish Health 2000 Study Soc Psychiatry Psychiatr Epidemiol 2006 41 1 11 17 10.1007/s00127-005-0011-5 16341826

[B15] Richardsen AM Martinussen M Factorial validity and consistency of the MBI-GS across occupational groups in Norway Int J Stress Manag 2005 12 289 297 10.1037/1072-5245.12.3.289

[B16] Derogatis L SCL-90-R. Administration, scoring & procedures. Manual-II 1992 Inc. CPR. Towson (MD)

[B17] Beck AT Steer RA Garbin M Psychometric properties of the Beck Depression Inventory: Twenty-five years of evaluation Clin Psychol Rev 1988 8 77 100 10.1016/0272-7358(88)90050-5

[B18] Grossi G Santell B Quasi-experimental evaluation of a stress management programme for female county and municipal employees on long-term sick leave due to work-related psychological complaints J Rehabil Med 2009 41 8 632 638 10.2340/16501977-0379 19565157

[B19] Heiden M Lyskov E Nakata M Sahlin K Sahlin T Barnekow-Bergkvist M Evaluation of cognitive behavioural training and physical activity for patients with stress-related illnesses: a randomized controlled study J Rehabil Med 2007 39 5 366 373 10.2340/16501977-0053 17549327

[B20] van der Klink JJ Blonk RW Schene AH van Dijk FJ Reducing long term sickness absence by an activating intervention in adjustment disorders: a cluster randomised controlled design Occup Environ Med 2003 60 6 429 437 10.1136/oem.60.6.429 12771395 PMC1740545

[B21] Lander F Friche C Tornemand H Andersen JH Kirkeskov L Can we enhance the ability to return to work among workers with stress-related disorders? BMC public health 2009 9 372 10.1186/1471-2458-9-372 19804632 PMC2765963

